# Correction to: The duration of caffeine treatment plays an essential role in its effect on sleep and circadian rhythm

**DOI:** 10.1093/sleepadvances/zpad023

**Published:** 2023-05-08

This is a correction to: Aishwarya Segu, Nisha N Kannan, The duration of caffeine treatment plays an essential role in its effect on sleep and circadian rhythm, *SLEEP Advances*, Volume 4, Issue 1, 2023, zpad014, https://doi.org/10.1093/sleepadvances/zpad014

In the originally published version of this manuscript, there were several errors in the graphical abstract.


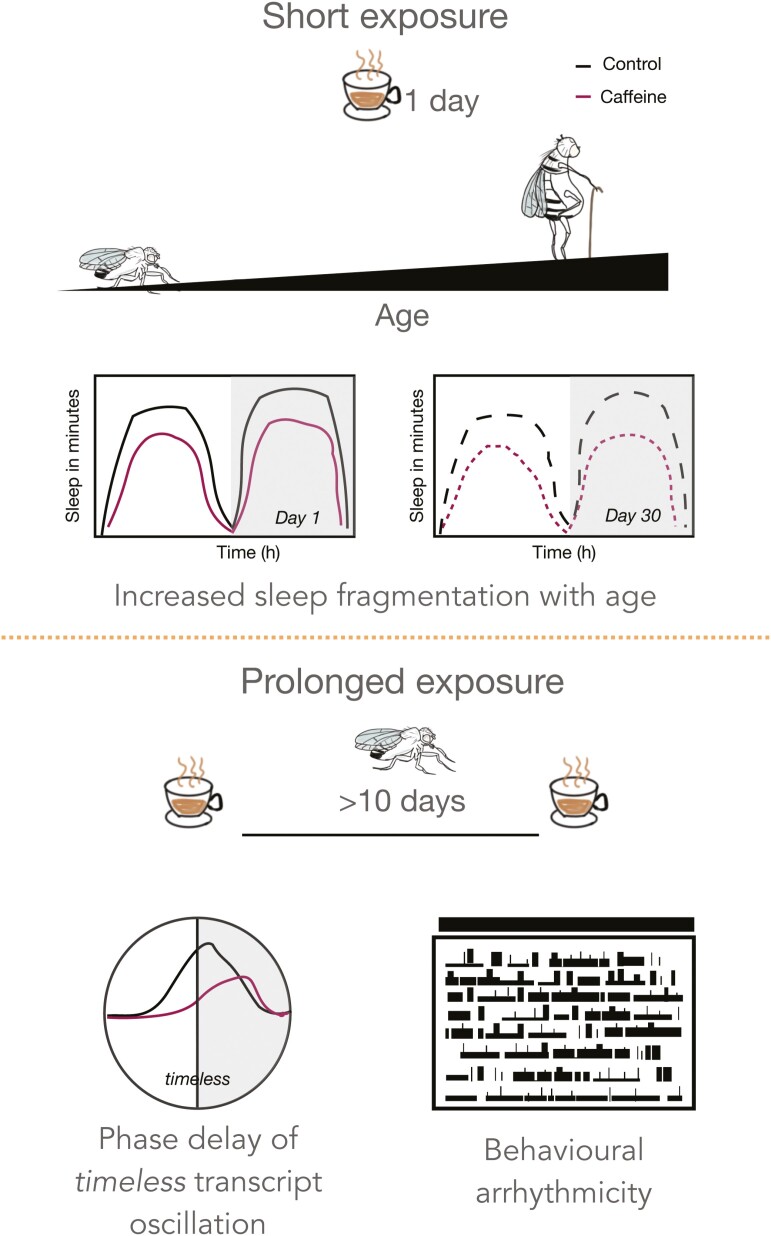


These errors have now been corrected.

