# Supplementary material for: The duration of caffeine treatment plays an essential role in its effect on sleep and circadian rhythm
Source: Sleep Adv. 2023 Feb 15;4(1):zpad014. doi: 10.1093/sleepadvances/zpad014 (PMC10108652; doi:10.1093/sleepadvances/zpad014)
Supplement: zpad014_suppl_Supplementary_Material [file zpad014_suppl_supplementary_material.pdf]

Supplementary Material for

The duration of caffeine treatment plays an essential role in its effect on sleep and circadian rhythm

Aishwarya Segu and Nisha N Kannan*

Chronobiology Laboratory, School of Biology, Indian Institute of Science Education and Research (IISER), Thiruvananthapuram, Kerala 695551, India

*Corresponding author. e-mail address: nishankannan@iisertvm.ac.in

Tel.: +0471-2778045

Figure S1

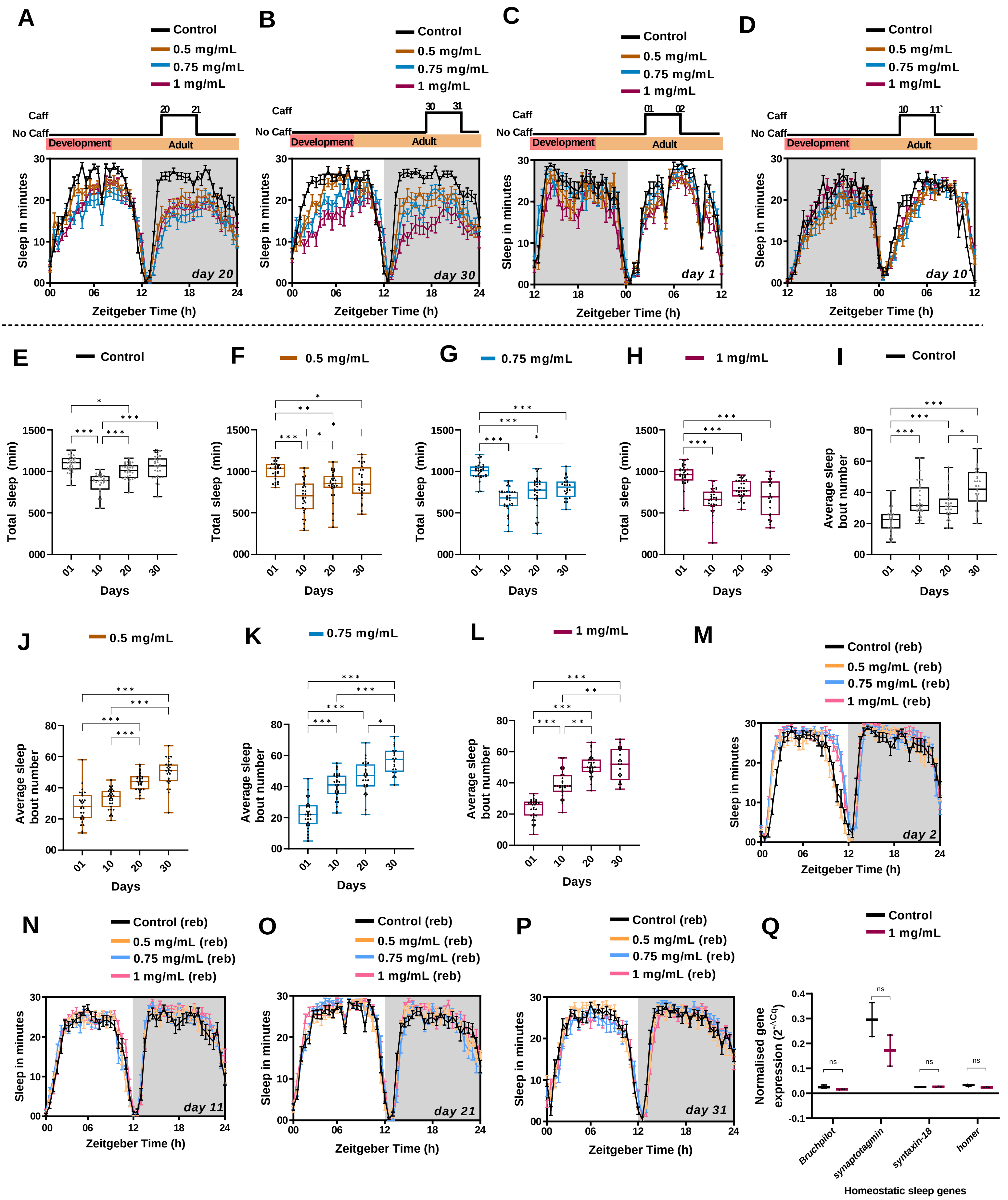

Figure S1: Effect of short exposure to caffeine on homeostatic sleep.
(A, B) Sleep in min for every 30 min over a period of 24h under LD is shown for 20 and 30 day old w^{1118} flies of control, 0.5 mg/mL, 0.75 mg/mL and 1 mg/mL under short exposure to caffeine. Schematic on top of the graph depicts short exposure to caffeine. For day 20 old flies the caffeine treatment was from day 20th to 21th and for 30 day old flies it was from 30th to 31sh day. (C, D) Sleep in min for every 30 min over a period of 24h under LD with nightime induction of caffeine (ZT 12) is shown for 1 and 10 day old w^{1118} flies of control, 0.5 mg/mL, 0.75 mg/mL and 1 mg/mL under short exposure to caffeine. Schematic on top of the graph depicts short exposure to caffeine. For day 1 old flies the caffeine treatment was from ZT 12 (Day 1) to ZT 12 (Day 2) and for 10 day old flies it was from ZT 12 (Day 10) to ZT 12 (Day 11). (E-H) Quantified total sleep in min for short exposure to caffeine with 0.5, 0.75 and 1 mg/mL caffeine concentration across 1, 10, 20 and 30 day old flies. (I-L) Quantified average sleep bout numbers for short exposure to caffeine with 0.5, 0.75 and 1 mg/mL caffeine concentration across 1, 10, 20 and 30 day old flies. (M-P) Sleep rebound in min for every 30 min over a period of 24h under LD is shown for 1, 10, 20 and 30 day old w1118 flies immediately after for 24h of short exposure to 0.5 mg/mL, 0.75 mg/mL and 1 mg/mL of caffeine. (Q) Quantified mRNA level for *bruchpilot*, *synaptotagmin*, *syntaxin-18* and *homer* of 10 day old flies under short term caffeine exposure to 1 mg/mL concentration.

Figure S2

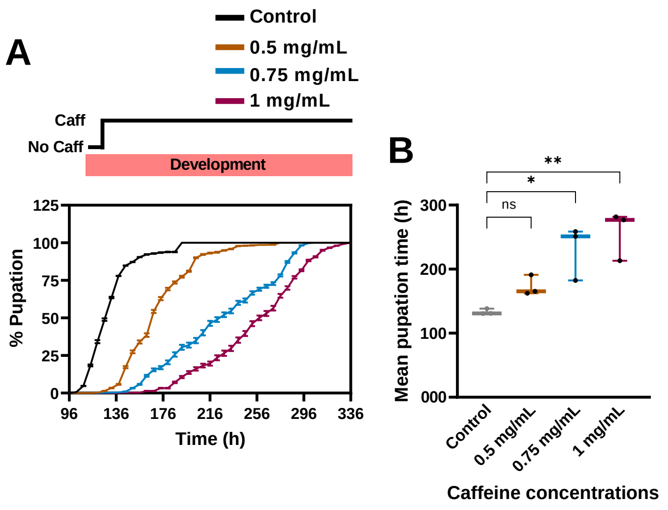

Figure S2: Effect of prolonged exposure to caffeine on sleep and development. (A-B) Percentage of pupation and mean pupation time in hr under different concentrations of prolonged caffeine treatment. Schematic given above the graph illustrates prolonged caffeine treatment during the larval stages. (C) Mean sleep bout length of 10 day old flies under 0.5, 0.75 and 1 mg/mL caffeine concentrations of prolonged caffeine treatment. No significant difference was observed in sleep bout length when compared to the control. Schematic on top of the graph illustrates the prolonged caffeine treatment protocol. From 1st instar larval stages till the end of the experiment the flies were provided with caffeine containing cornmeal dextrose medium. (D) Food intake post prolonged caffeine exposure for 10 day old flies. Food intake was assessed from ZT 01-04 using CAFE in the absence of caffeine during the assay. No significant difference was observed in food intake when compared to the control. (E) Sleep latency of 10 day old flies with 0.5mg/mL, 0.75 mg/mL and 1 mg/mL of prolonged caffeine treatment. No significant difference was observed in sleep latency when compared to the control. (F) Quantified total sleep in min from ZT 23-24 for 10 day old w^{1118} flies under 0.5, 0.75 and 1 mg/mL of prolonged caffeine exposure (Kruskal-Wallis Test followed by Dunn's multiple comparisons control v/s 0.75 mg/mL $p< 0.01$, control v/s 0.75 mg/mL $p< 0.001$). (G) Quantified total sleep in min from ZT 03-04 for 10 day old w^{1118} flies under 0.5, 0.75 and 1 mg/mL of prolonged caffeine exposure (Kruskal-Wallis Test followed by Dunn's multiple comparisons control v/s 1 mg/mL $p< 0.05$).

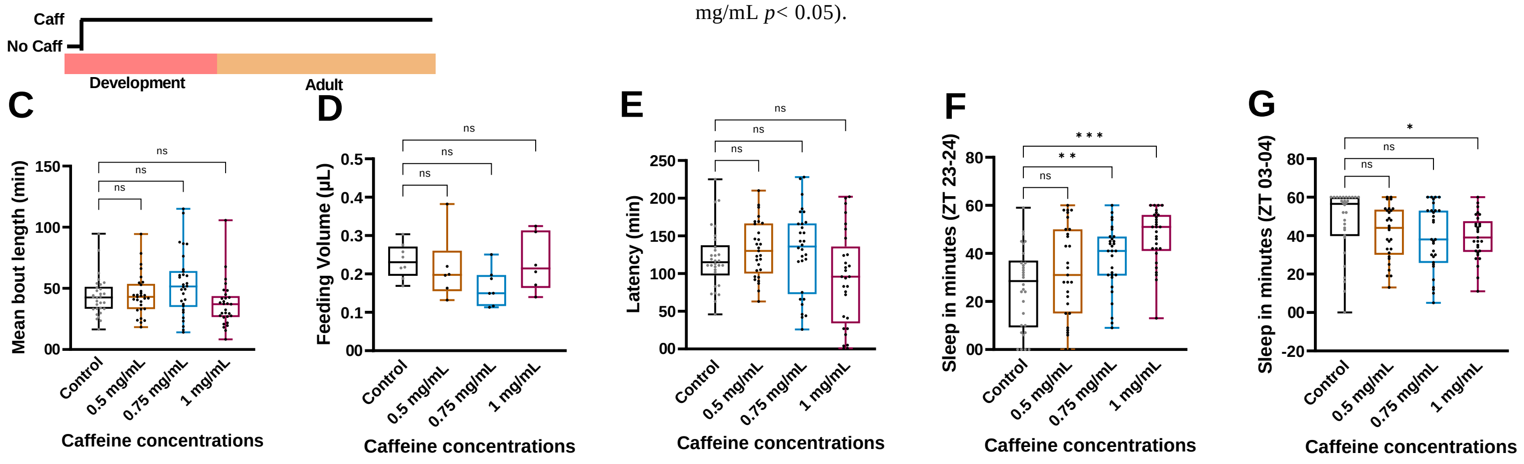

Figure S3

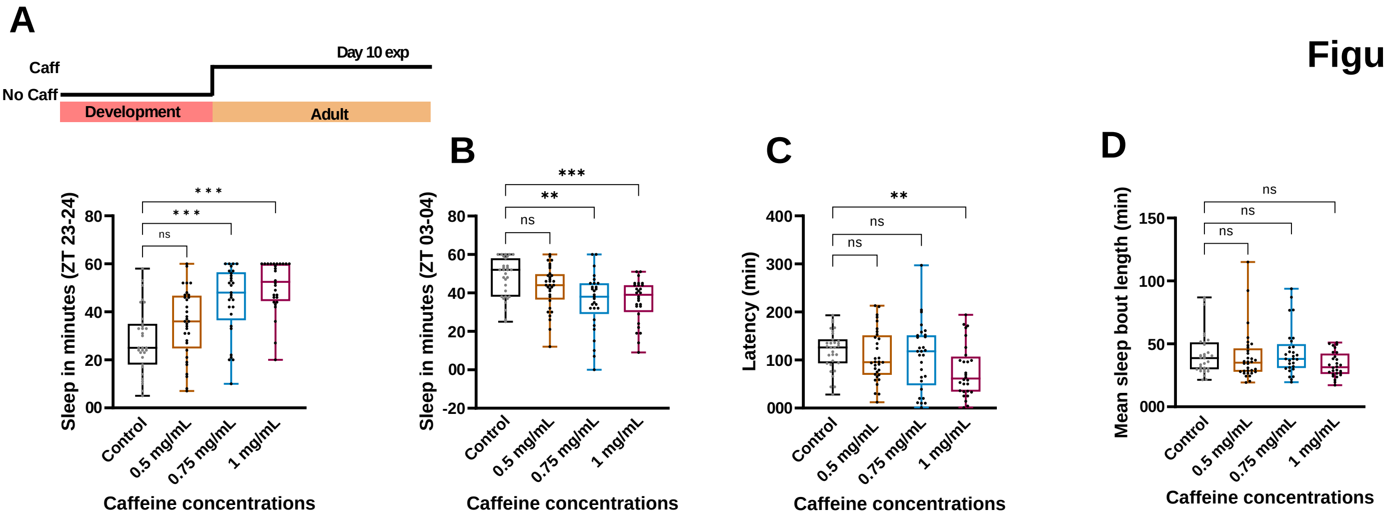

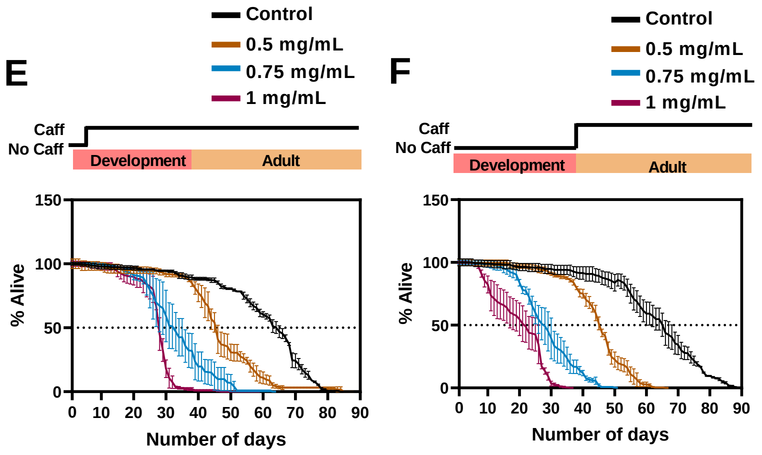

Figure S3: Effect of adult stage specific caffeine treatment on sleep. (A) Quantified total sleep in min from ZT 23-24 for 10 day old w^{1118} flies under 0.5, 0.75 and 1 mg/mL of prolonged caffeine exposure (Kruskal-Wallis Test followed by Dunn's multiple comparisons control v/s 0.75 mg/mL $p< 0.01$, control v/s 0.75 mg/mL $p< 0.001$). (B) Quantified total sleep in min from ZT 03-04 with adult stage specific caffeine treatment for 10 days in w^{1118} flies under 0.5, 0.75 and 1 mg/mL caffeine concentration (Kruskal-Wallis Test followed by Dunn's multiple comparisons; control v/s 0.75 mg/mL $p<0.001$; control v/s 1 mg/mL $p< 0.001$). (C) Sleep latency of day 10 old flies with 0.5mg/mL, 0.75 mg/mL and 1 mg/mL caffeine concentration under adult stage specific caffeine treatment. 1 mg/mL caffeine concentration showed a decrease in sleep latency when compared to the control (Kruskal-Wallis Test followed by Dunn's multiple comparisons control v/s 1 mg/mL $p< 0.05$). (D) Mean sleep bout length of 10 day old flies under 0.5, 0.75 and 1 mg/mL adult-stage specific caffeine treatment. No significant difference was observed in sleep bout length when compared to the control. (E) Lifespan of w^{1118} flies under different concentrations of prolonged caffeine treatment. Percentage of flies alive is plotted on the y-axis and time in days is plotted on the x-axis. It is represented with the schematics for prolonged caffeine treatment. (F) Lifespan of w^{1118} flies under different concentrations of adult stage specific caffeine treatment. Percentage of flies alive is plotted on the y-axis and time in days is plotted on the x-axis. It is represented with the schematics for adult stage caffeine treatment.

A B **Figure S4**

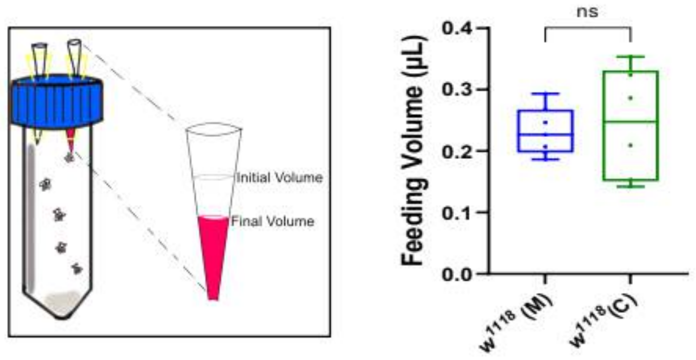

Figure S4: Capillary feeding assay using micro tips

A modified protocol of CAFE with micro tips instead of capillary tubes was used in the manuscript to analyse food intake. Micro capillary tubes and micro tips work on the same principle, and the latter comes at a very cheaper rate. The capillary tubes are cylindrical in shape whereas the micro tips are conical with two open ends. The volume of the open ended cone is as follows:

$$\mathbf{V = 1/3\,\pi\,\{(r_1)^2 + (r_1 r_2) + (r_2)^2\} \times h}$$

According to the above formula three variables namely radii - r_1 and r_2 and height - h of the cone required for volume calculation. That meant after every feeding assay one needs to cut the tip to measure the radius of the cone, which is not feasible and error prone. To overcome this we used the two model of the cone and using the two-point line equation namely $\mathbf{y_1 - y_2 = m(x_1 - x_2) + c}$. Based on our conditions the equation is modified as $\mathbf{h_2 - h_1 = m(r_2 - r_1) + c}$. As we provide a fixed volume (10µL) at the start of every assay this was r_1, r_2 is the radius at the base of the tip. Both of these radii were calculated by cutting the tip and the exact radius was measured using optical microscope. The corresponding height h_1 was measured using vernier caliper. The height h_2 which is the base of the tip is always zero. From these above values we used the above 2-point line equation to calculate the slope of the line. In this way, after every feeding assay we were able to measure the new radius by using the slope. Further, the remaining volume in the tip was calculated and this volume was subtracted by the initial volume (10 µL) to get the volume fed by the flies. After the volume calculations to validate our method we performed 3h feeding assay using both micro-tips and micro-capillary tubes in *w*^{1118} male flies. The volume fed by flies in both methods showed no significant difference indicating that our methodology can be used for CAFE (Figure S4B). With this validation we conducted the rest of the feeding assays with our modified protocol.

Table S1: List of primers used for quantitative RT-PCR.

Gene	Forward Primer	Reverse Primer
rp49	GCTAAGCTGTCGCACAAA	TCCGGTGGGCAGCATGTG
timeless	CCGTGGACGTGATGTACCGCAC	CGCAATGGGCATGCGTCTCTG
homer	GAACAACCGATTTTCACTGC	GAGCTGTCGTAGAAGGAAGCTAAC
synaptotagmin	CTGAGTCCGGTCTTCAACGAG	ACACGAGCGTCTTGTTCATGG
bruchpilot	GCAGTCCATACTACCGCGAC	TTGGATAGTCCATGGCATGGG
Syntaxin 18	GTGTGCCAACAAGATCACTCGC	GCGTGTAAAAGGTGACGAACTTC

Table S2: Statistical details of effect of prolonged caffeine treatment on activity rest rhythm of 10 day old flies.

The table provides details of Zeitgeber Time (h) where a significant difference in activity rest rhythm was observed in each of the caffeine concentrations (0.5, 0.75, 1 mg/mL) when compared to the control.

**Caffeine concentration: 0.5 mg/mL (comparison with control)**		
**Zeitgeber Time (h)**	**Summary**	**Adjusted *p* Value**
ZT 00	***	<0.001
ZT 10.75	**	0.003
ZT 11	***	<0.001
ZT 11.25	**	0.002
ZT 12	**	0.007
ZT 12.75	***	<0.001
ZT 13 and 13.25	***	<0.001
**Caffeine concentration: 0.75 mg/mL (comparison with control)**		
ZT 23.25	*	0.01
ZT 12.50,12.75 and 13	***	<0.001
ZT 13.25	**	0.001
**Caffeine concentration: 1 mg/mL (comparison with control)**		
ZT 22	*	0.03
ZT 22.50 and 22.75	***	<0.001
ZT 23	**	0.003
ZT 23.25 to 00.25	***	<0.001
ZT 00.50	*	0.02
ZT 10.50 to 12	***	<0.001
ZT 12.75 to 14	***	<0.001
ZT 14.50	*	0.02

Two-way ANOVA followed by Tukey's post hoc HSD showing Zeitgeber time points (h) at which 0.5, 0.75 and 1 mg/mL caffeine treated flies showed significant difference in activity rest rhythm when compared to the control.

Table S3: Metacycle outcome for *timeless* transcript oscillation.

	**JTK_pvalue**	**JTK_BH.Q**	**JTK_period**	**JTK_adjphase**	**Meta2d_Base**	**Meta2d_AMP**
control	0.000115	0.000230	20	12	0.0098117	0.00932
1 mg/mL	0.00754	0.00454	24	16	0.00327	0.00699

The table contains the outcome of JTK analysis performed using metacycle meta2d algorithm followed by combined metacyle analysis. It also provides the *p*-value for the *timeless* transcript in control and 1 mg/mL which defines the oscillation of the transcript. Further, it also contains the phase and period of the transcript under each condition.

Table S4: Statistical details of effect of adult stage specific caffeine treatment on activity rest rhythm.

The table provides details of Zeitgeber Time (h) where a significant difference in activity rest rhythm was observed in each of the caffeine concentrations (0.5, 0.75, 1 mg/mL) when compared to the control.

**Caffeine concentration: 0.5 mg/mL (comparison with control)**		
**Zeitgeber Time (h)**	**Summary**	**Adjusted *p* Value**
ZT 12.75 to 13.50	***	<0.001
**Caffeine concentration: 0.75 mg/mL (comparison with control)**		
ZT 00.25	**	0.01
ZT 10.75	*	0.04
ZT 11 to 13.75	***	<0.001
**Caffeine concentration: 1 mg/mL (comparison with control)**		
ZT 23.50	*	0.02
ZT 23.75	**	0.005
ZT 00 to 00.75	***	<0.001
ZT 02.75	*	0.04
ZT 10.50	*	0.03
ZT 10.75 to 12.50	***	<0.001
ZT 13.25	***	<0.001
ZT 13.50	***	<0.001
ZT 13.75	*	0.02

Two-way ANOVA followed by Tukey's post hoc HSD showing Zeitgeber time points (h) at which 0.5, 0.75 and 1 mg/mL caffeine treated flies showed significant difference in activity rest rhythm when compared to the control.

Table S5: Statistical details of effect of adult stage caffeine treatment on young flies on activity rest rhythm.

The table provides details of Zeitgeber Time (h) where a significant difference in activity rest rhythm was observed in each of the caffeine concentrations (0.5, 0.75, 1 mg/mL) when compared to the control.

**Caffeine concentration: 0.5 mg/mL (comparison with control)**		
**Zeitgeber Time (h)**	**Summary**	**Adjusted *p*-value**
ZT 09.50	***	<0.001
ZT 09.75 to 11	***	<0.001
ZT 12.50	**	0.006
ZT 12.75	***	<0.001
**Caffeine concentration: 0.5 mg/mL (comparison with control)**		
ZT 09.75 and 10	***	<0.001
ZT 10.25	**	0.001
ZT 10.50 to 11.50	***	<0.001
ZT 12.50 to 13	***	<0.001
ZT 13.25	**	0.003
**Caffeine concentration: 1 mg/mL (comparison with control)**		
ZT 00.25 to 01	***	<0.001
ZT 01.25	**	0.002
ZT 22.25	**	0.008
ZT 22.25 to 13.50	***	<0.001

Two-way ANOVA followed by Tukey's post hoc HSD showing Zeitgeber time points (h) at which 0.5, 0.75 and 1 mg/mL caffeine treated flies showed significant difference in activity rest rhythm when compared to the control.
